# Wanted: role models - medical students’ perceptions of professionalism

**DOI:** 10.1186/1472-6920-12-115

**Published:** 2012-11-15

**Authors:** Anna Byszewski, Walter Hendelman, Caroline McGuinty, Geneviève Moineau

**Affiliations:** 1 Professor of Medicine, Division of Geriatric Medicine, University of Ottawa, Ottawa, Canada; 2 Professor Emeritus, University of Ottawa, Ottawa, Canada; 3 Postgraduate Training, Internal Medicine, University of Toronto, Toronto, Canada; 4 Vice President, Education, Association of Faculties of Medicine of Canada, Toronto, Canada; 5 The Ottawa Hospital, Civic Campus, 1053 Carling Avenue, Box 678, Ottawa, ON, Canada

**Keywords:** Professionalism, Curriculum, Undergraduate medical education, Learning environment

## Abstract

**Background:**

Transformation of medical students to become medical professionals is a core competency required for physicians in the 21^st^ century. Role modeling was traditionally the key method of transmitting this skill. Medical schools are developing medical curricula which are explicit in ensuring students develop the professional competency and understand the values and attributes of this role. The purpose of this study was to determine student perception of professionalism at the University of Ottawa and gain insights for improvement in promotion of professionalism in undergraduate medical education.

**Methods:**

Survey on student perception of professionalism in general, the curriculum and learning environment at the University of Ottawa, and the perception of student behaviors, was developed by faculty and students and sent electronically to all University of Ottawa medical students. The survey included both quantitative items including an adapted Pritzker list and qualitative responses to eight open ended questions on professionalism at the Faculty of Medicine, University of Ottawa. All analyses were performed using SAS version 9.1 (SAS Institute Inc. Cary, NC, USA). Chi-square and Fischer’s exact test (for cell count less than 5) were used to derive p-values for categorical variables by level of student learning.

**Results:**

The response rate was 45.6% (255 of 559 students) for all four years of the curriculum. 63% of the responses were from students in years 1 and 2 (preclerkship). Students identified role modeling as the single most important aspect of professionalism. The strongest curricular recommendations included faculty-led case scenario sessions, enhancing interprofessional interactions and the creation of special awards to staff and students to “celebrate” professionalism. Current evaluation systems were considered least effective. The importance of role modeling and information on how to report lapses and breaches was highlighted in the answers to the open ended questions.

**Conclusions:**

Students identify the need for strong positive role models in their learning environment, and for effective evaluation of the professionalism of students and teachers. Medical school leaders must facilitate development of these components within the MD education and faculty development programs as well as in clinical milieus where student learning occurs.

## Background

The core values associated with patient care, including compassion, empathy, altruism and honesty, are presently understood under the term medical professionalism. In 1999 the Association of American Medical Colleges (AAMC) recommended that all medical schools include professionalism in the core curriculum of medical education [1]. The Royal College of Physicians and Surgeons (Canada) in 2000 developed the core competencies, CanMEDS, which were revised in 2005, with ‘The Professional’ as one of the core physician competencies and this role includes ethics, professionalism as now understood, and care of self [2]. Jha and colleagues [3] in an interview study of medical educators, students, doctors, allied health professionals and lay professionals explored what are the key professionalism themes. Their qualitative analysis yielded seven themes: compliance to values, patient access, doctor-patient relationship, demeanor, professional management, personal awareness and motivation.

Traditionally, these behaviors were transmitted by respected role models. Today, with the apprenticeship model being replaced with a competency based model, this approach alone is no longer sufficient. More than a decade ago Cruess and Cruess [4] described the need for the explicit teaching of the definition and values of professionalism, and that institutional leadership and support is needed to demonstrate its fundamental significance [5]. Level specific activities and an opportunity for reflection must exist hand in hand with role modeling.

Almost 100 years after Flexner’s report on medical education, which noted that scientific medicine was sadly deficient in cultural and philosophic background, we are still struggling, as recently commented on by Cooke [6]. Medical curricula must emphasize not only the science of medicine, that which is only evidence based, but also the more subtle but so essential art of medicine. Current “ossified curricular structures, a persistent focus on the factual minutiae of today’s knowledge base”, and faculty burdened with workload and aspiring efficiency, can threaten essential values that make for an inquisitive and empathic physician [6]. Paralleling this work has been the deeper understanding of the experiences which shape what medical students learn about professionalism during classwork and in the course of clinical care. This includes attitudes and behaviors which often contradict moral and ethical values conveyed by the formal curriculum, the so-called hidden curriculum [7-9]. Medical students often struggle to develop a professional identity, while surviving the demanding academic and clinical challenges in a complex and ambiguous environment [10]. The Association of Faculties of Medicine of Canada – Future of Medical Education in Canada (FMEC) Project included addressing the hidden curriculum as one of its ten recommendations [10]. The LCME (Liaison Committee on Medical Education) accreditation standard applies also for Canadian and American medical schools. Standard MS-31-A states: “a medical education program must ensure that its learning environment promotes the development of explicit and appropriate professional attributes in its medical students (i.e., attitudes, behaviors, and identity)” [11]. Studies by Papadakis and others have shown an association between disciplinary actions of practicing physicians by medical boards with unprofessional behavior in medical school [12-14]. It is thus critical that each training program has an obligation to ‘teach’ and evaluate professionalism amongst its medical students.

Van Mook et al provide a Dutch perspective on assessment of professional behaviors and emphasize that assessment drives learning [15]. This model combines both a formative and summative approach. To conquer the reluctance to address minor lapses, practical guidelines are provided to tutors. A framework for confrontation, feedback and remediation is provided. They emphasize early identification of lapses as being critical in avoiding behavior becoming refractory to change.

Stern and Papadakis have set out a realistic strategy for a professionalism program, including the following: setting expectations (codes and charters), providing experience (role models) and evaluating outcomes [16].

Our initial MD undergraduate professionalism program at the University of Ottawa, Ontario, Canada was implemented in 2002. As part of a complete curriculum renewal process, the professionalism leadership was charged with reviewing the professionalism program and developing recommendations for future implementation. Part of this review included a survey of current students to determine their perceptions.

The professionalism program at the time of the survey included an Introductory session to Professionalism, given to the first year students in the first week of the orientation week (led by senior students), and a formal “White Coat” ceremony attended by the Dean, faculty representatives and officials, with reading and signing by the students of the University of Ottawa Declaration of Professionalism at the end of the week [17]. In the first two years (preclerkship), twice yearly (total of 4 sessions) faculty-led small group case scenario sessions were organized for discussions of hypothetical cases on attributes of professionalism. Formal activities in the clerkship (last two years) consisted of a lecture during the introduction to clerkship program.

## Objective of the survey

The purpose of this work is to determine student perception of professionalism, the professionalism curriculum, the current learning environment and suggestions for improvement.

## Methods

A survey was developed by faculty and medical student members of the professionalism review team, as part of a curriculum review. The survey incorporated a series of questions about what behaviors students considered as being unprofessional, including 12 questions based on the Pritzker model [18] (used with permission) and quantitative and qualitative components. The qualitative, open ended eight questions were placed at the end of the survey and asked about what professionalism meant to the students, how they viewed the current curriculum, and how it could be improved. The survey was sent to all University of Ottawa medical students in December 2006, with two email reminders. The Ottawa Research Ethics Board had no objections to the content of the survey.

## Data analysis

Most of the questions consisted of 4 point Likert scale which in the analysis was collapsed into two overall categories: agree and disagree (except Table 1). All analyses were performed with SAS version 9.1 (SAS Institute Inc., Cary, NC, USA.). Chi-square test and Fisher’s exact test (for cell count less than 5) were used to derive p-values for categorical variables by level of student year of training. We defined the significance level as p < 0.05. A limited review of the qualitative responses for themes was done, given this was an electronic survey that generated only a number of responses, comparing the preclerkship to clerkship student responses.

**Table 1:** Student perceptions of the University of Ottawa professionalism program effectiveness by year of study

**Question asked: the following are effective components of the professionalism program:**		**Year1 (n = 74)**	**Year2 (n = 87)**	**Year3 (n = 36)**	**Year4 (n = 58)**	**Total (n = 255)**	**P-value**
**Clinical interactions**	Disagree	4.1%	10.3%	8.3%	6.9%	7.5%	0.501
	Agree	95.9%	89.7%	91.7%	93.1%	92.5%	
Case scenario sessions *	Disagree	5.4%	21.8%	19.4%	22.4%	16.9%	0.019
	Agree	94.6%	78.2%	80.6%	77.6%	83.1%	
**The formal professionalism course**	Disagree	14.9%	27.6%	22.2%	22.4%	22.0%	0.285
	Agree	85.1%	72.4%	77.8%	77.6%	78.0%	
**Orientation week**	Disagree	24.3%	35.6%	30.6%	32.8%	31.0%	0.475
	Agree	75.7%	64.4%	69.4%	67.2%	69.0%	
Presentations *	Disagree	20.3%	34.5%	44.4%	32.8%	31.4%	0.057
	Agree	79.7%	65.5%	55.6%	67.2%	68.6%	
Evaluations of professionalism *	Disagree	23.0%	63.2%	55.6%	36.2%	44.3%	<.001
	Agree	77.0%	36.8%	44.4%	63.8%	55.7%	

(*Differences by year are significant at p < .05).

## Results

The response rate was 45.6%, with 255 responses from a possible 559. The breakdown of the 255 respondents was: year 1, 29%, year 2, 34%, year 3, 14% and year 4, 23%. When asked regarding their confidence about understanding professionalism, 97% of students answered in the affirmative. Some examples of responses include from year I: “Demonstrating moral and ethical behavior, and treating peers, co-workers, and patients with the proper amount of respect.” From year II: “Interacting with colleagues, patients, families and fellow humans in an ethical and dignified way… to ensure that their rights and privileges are protected, while not compromising one’s own beliefs and values.” and from year III: “Professionalism means treating patients equally and with the utmost respect; it means always maintaining patient confidentiality; it means being responsible and accountable for my actions; it means showing compassion; it means striving to become a competent and well-rounded physician.” It was evident that some of the preclerkship students knew what were the attributes of professionalism (respect, honesty, etc.) but the students in higher years were able to elaborate more on the context of these attributes as they relate to clinical interactions with patients and co-workers.

When asked how to improve the professional curriculum, preclerkship students focused more on discussions around integrating professionalism discussions into PBL sessions, whereas clerkship students highlighted the opportunity to develop professional behavior during clinical encounters. Clerkship students were more likely to comment on the importance of role modeling, small group discussions or reflective narratives, and the need to have a clear process to address lapses in professionalism, including the reporting process. Replies to this question “Please suggest ways the medical school may help you develop your professionalism” provided important insights such as: “strong role models as well as addressing unprofessional behavior of peers” and “encourage greater professionalism among staff during clerkship”.

The components of the professionalism program are compared in Table 1. Overall, 78% agreed that the current formal professionalism course was effective. The single most important effective component of learning professionalism (92.5%) as determined by students in all four years was “clinical interactions–role modeling”. Case scenarios were next with agreement by 83.3% of students. These were faculty led small group sessions. The current evaluation of professionalism was considered ineffective by 44.3% of respondents. It is important to note however, that Year 1 students were much more likely to find the curriculum and evaluations of professionalism to be effective than students in Years 2 through 4, although by year 4 there was a trend upwards. Students are exposed to case scenario sessions as well as evaluations of PBL sessions from the very beginning of the first year of the curriculum.

When the surveyed students were presented with a list of student behaviors (Table 2) from the Pritzker list, most students (both in preclerkship and clerkship) agreed that skipping classes, inebriation, and late arrival were all unprofessional. Most students (66-86%) responded that leaving lectures earlier, not filling out mandatory evaluations, skipping a non-compulsory class or lab, and checking email during lecture, were not unprofessional behaviors.

**Table 2:** Perspective on behaviors based on the Pritzker list (used with permission, adapted)

**Q: Are these behaviors unprofessional for a medical student?**	**Yes**	**No**
Repetitive late arrival to class	192 (75%)	63 (25%)
Checking email, sports, MSN, or news on laptop during lecture	97 (38%)	158 (62%)
Taking food from talks that you are not attending	185 (73%)	70 (27%)
Signing another student in for a required class	230 (90%)	25 (10%)
Leaving lecture before its conclusion	75 (29%)	180 (71%)
Leave class if the lecturer's quality is lower than expected	110 (43%)	145 (57%)
Switch sessions from an assigned room to another room	114 (45%)	141 (55%)
Skip a class or lab in which attendance is required	205 (80%)	50 (20%)
Skip a class or lab in which attendance is not required	37 (15%)	218 (85%)
Inebriation at school events	194 (76%)	61 (24%)
Skipping required school events (e.g. orientation, retreat).	164 (64%)	91 (36%)
Combining PBL 2 with PBL 1 of the next week	16 (6%)	239 (94%)
Discuss issues relating to religion or politics on the Listserv	111 (44%)	144 (56%)
Give review sessions geared toward exam questions which the Prof. has shared at special reviews	35 (14%)	220 (86%)
Using a colleague's power point presentation to give during a clerkship teaching session	132 (52%)	123 (48%)
Leaving a written sign-out for a colleague taking over on call instead of waiting to present verbally	195 (76%)	60 (24%)
Not filling out the student questionnaire	48 (19%)	207 (81%)

When asked to rate the behaviors associated with professionalism, the most highly ranked were: Respect 66.7%, Integrity 59.6% and Honesty 56.5% (Table 3a). The lowest ranked were Compassion 5.9%, Dedication 4.7% and Empathy 4.3%*.* When asked to choose which of the same attributes were not adequately emphasized by the faculty, the most frequently chosen included Self-improvement 22.7%, Compassion 16.9% and Empathy 16.9% (Table 3b). This suggests that if behaviors (such as empathy, compassion and self improvement) are not role modeled by, and paid attention to by faculty, they will also be least likely to be valued by the students themselves. The open ended questions suggested that students tended to understand these terms better (the attributes of professionalism) in clerkship vs preclerkship. However this is not as evident in the responses to the list of attributes (Tables 3a and 3b) as there did not appear to be much difference when comparing the response from preclerkship to clerkship, except for accountability increasing in significance in clerkship. This could have been explored in more depth if we had the opportunity to conduct more in depth interviews with the students.

**Table 3:** Student perception of behaviors associated with professionalism

	**Preclerkship (n = 161)**	**Clerkship (n = 94)**	**Total (n = 255)**
**a. Student perception of behaviors associated with professionalism**			
**Respect**	**113 (70.2%)**	**57 (60.6%)**	**170 (66.7%)**
**Integrity**	**93 (57.8%)**	**59 (62.8%)**	**152 (59.6%)**
**Honesty**	**94 (58.4%)**	**50 (53.2%)**	**144 (56.5%)**
**Responsibility**	75 (46.6%)	46 (48.9%)	121 (47.5%)
**Accountability**	54 (33.5%)	39 (41.5%)	93 (36.5%)
**Self-improvement**	14 ( 8.7%)	11 (11.7%)	25 (9.8%)
**Altruism**	14 ( 8.7%)	8 (8.5%)	22 (8.6%)
**Compassion**	9 (5.6%)	6 (6.4%)	15 (5.9%)
**Dedication**	8 (5.0%)	4 (4.3%)	12 (4.7%)
**Empathy**	9 (5.6%)	2 (2.1%)	11 (4.3%)
**b. Student perception of behaviors not adequately emphasized by faculty**			
**Self-improvement**	**37 (23.0%)**	**21 (22.3%)**	**58 (22.7%)**
**Compassion**	**28 (17.4%)**	**15 (16.0%)**	**43 (16.9%)**
**Empathy**	**31 (19.3%)**	**12 (12.8%)**	**43 (16.9%)**
**Accountability**	21 (13.0%)	18 (19.1%)	39 (15.3%)
**Altruism**	26 (16.1%)	11 (11.7%)	37 (14.5%)
**Dedication**	25 (15.5%)	9 (9.6%)	34 (13.3%)
**Respect**	17 (10.6%)	9 (9.6%)	26 (10.2%)
**Responsibility**	11 (6.8%)	12 (12.8%)	23 (9.0%)
**Honesty**	8 (5.0%)	10 (10.6%)	18 (7.1%)
**Integrity**	7 (4.3%)	7 (7.4%)	14 (5.5%)

Student perceptions of ways to improve the curriculum are represented in Table 4. The strongest recommendation is the enhancement of inter-professional interactions (58.4% agreement). Guest lectures was considered important by 57% and the creation of professionalism awards to faculty and students was considered important by 42% and was rated statistically higher by students in the clerkship years.

**Table 4:** Student opinion regarding potential improvements to curriculum

		**Year 1 n = 74**	**Year 2 n = 87**	**Year 3 n = 36**	**Year 4 n = 58**	**Total n = 255**	**P-value**
**Inter-professional interactions**	Agree	70.3%	57.5%	41.7%	55.2%	58.40%	0.123
	No opinion	20.3%	26.4%	30.6%	27.6%	25.50%	
	Disagree	9.5%	16.1%	27.8%	17.2%	16.10%	
**Guest lectures**	Agree	58.1%	55.2%	38.9%	70.7%	57.30%	0.11
	No opinion	24.3%	26.4%	33.3%	20.7%	25.50%	
	Disagree	17.6%	18.4%	27.8%	8.6%	17.30%	
Awards to staff and students*	Agree	35.1%	35.6%	50.0%	55.2%	42.00%	0.039
	No opinion	27.0%	26.4%	22.2%	6.9%	21.60%	
	Disagree	37.8%	37.9%	27.8%	37.9%	36.50%	
**Publication of disciplinary measures**	Agree	41.9%	34.5%	47.2%	41.4%	40.00%	0.126
	No opinion	24.3%	20.7%	36.1%	27.6%	25.50%	
	Disagree	33.8%	44.8%	16.7%	31.0%	34.50%	
**More disciplinary measures for infractions**	Agree	31.1%	33.3%	50.0%	34.5%	35.30%	0.377
	No opinion	35.1%	29.9%	19.4%	37.9%	31.80%	
	Disagree	33.8%	36.8%	30.6%	27.6%	32.90%	
**Professionalism web-page**	Agree	28.4%	43.7%	22.2%	31.0%	33.30%	0.261
	No opinion	39.2%	32.2%	47.2%	36.2%	37.30%	
	Disagree	32.4%	24.1%	30.6%	32.8%	29.40%	
**Special interest group on professionalism**	Agree	35.1%	20.7%	19.4%	25.9%	25.90%	0.163
	No opinion	50.0%	47.1%	50.0%	48.3%	48.60%	
	Disagree	14.9%	32.2%	30.6%	25.9%	25.50%	

(*Differences by year are significant at p < .05).

In addition 38% of students had witnessed or been part of an exemplary demonstration of professionalism. However 64% responded that they had witnessed some form of lapse of professionalism as a student in the MD program.

## Discussion

Students have great insights on their perceived needs with regard to their education*.* Most of the students reported that our current professionalism curriculum was effective. They prefer real cases discussed by clinical faculty to lecture based learning. Students want professionalism to have a prominent place at their school but not necessarily in their didactic curriculum. When compared to other areas of the curriculum, our students consider professionalism content as excessive and not fully effective (Table 1). Close to one third of the students found that the professionalism presentations and orientation week were ineffective. Formal activities were also seen as ineffective by close to one forth. This is also the perception of students across the country regarding their own programs [19] as the AAMC Canadian Medical School Graduation Questionnaire which is conducted across Canada at completion of medical school, found that 24% of all students (and 30% at the University of Ottawa) found the curriculum excessive. Leo and Eagen have studied why students “cringe” and feel patronized when the topic of professionalism is discussed in their curriculum [20]. They emphasize that to foster credibility, programs must promote and adhere to the same principles at all levels of training and position.

The University of Chicago Pritzker School of Medicine developed an institution-wide Roadmap to Professionalism, a program to increase awareness of medical professionalism [18]. It included the student survey using the Pritzker list of behaviors, which we used with permission. Over time, behaviors that the students previously considered unprofessional, became increasingly more acceptable as students progressed in their training, indicating some erosion of values. Our students also did not consider their own behaviors from the Pritzker list (Table 2), such as emailing during class or leaving class early, as unprofessional. This may be possibly related to student comfort with multi-tasking and self-directed learning, but it also suggests that our current generation of learners may have a different perception of professionalism as it relates to specific behaviors [21,22]. This raises the importance for teachers to clearly identify and make explicit the expectations of medical students as they interact in classroom and clinical settings.

In addition, students in our study identified that evaluation of professionalism was often seen as ineffective. Evaluation of professionalism is an acknowledged ‘Achilles heel’ in the efforts to develop a complete professionalism program. Ginsburg and collaborators have shown that behavior is contextual: that the student’s and the evaluator’s perception often determine whether the unprofessional behavior is actually recorded or reported [23-25]. In a study with faculty responding to five videotaped scenarios in which students were placed in professionally challenging situations, there was a general lack of agreement of how students should act [25]. There was a lack of a shared standard for professional behaviour in students, and the authors emphasize how context drives behaviour, and needs to be considered in professionally challenging situations, beyond the student’s apparent behaviour.

According to our students, role modeling continues to be the single most important component of the medical school experience as it relates to professionalism and the development of professional identity. In general, the attributes not highly emphasized by faculty are not highly valued by students (Tables 3a and 3b). Recent publications speak to the fact that empathy can be taught and role modeled [26,27] and yet this was one of the attributes least emphasized by faculty in our study, and the least highly ranked by the students. Our findings are similar to those of Brainard and Brislen who collected students’ experiences from five American medical schools, and found several barriers to medical professionalism education which included unprofessional conduct by medical educators, substandard professional behavior accepted in exchange for efficiency, and institutions lacking whistleblower protection [28]. Kenny et al. have stated that role modeling is an untapped educational resource that needs to be emphasized in faculty development initiatives [29].

Students from our school and all schools across the country continue to experience unprofessional behaviors in pre-clinical and clinical environments [19]. This is part of the “hidden curriculum” which in defined as a set of values which contradicts those of the formal curriculum [8,9]. Work from Hafferty suggests that the learning environment needs to reflect the professionalism curriculum, so that students can benefit from consistent education, clear expectations and assessment. The issue of the learning environment is one that must be addressed by medical schools, their affiliated institutions and all their distributed settings [11].

The McGill physicianship program initiated by Cruess and Cruess has put the principles that the cognitive base of professionalism must be taught explicitly [30], with a special emphasis on the social contract [31], into action [32], and is accompanied by faculty development [33]. The importance of faculty development cannot be overemphasized in this context.

There are several possible routes to improving the ‘culture of professionalism’ within a faculty or institution. A survey of Canadian faculties undertaken by the authors (AB, WH)in the same year indicated that most medical schools had a professionalism curriculum in place for the ‘pre clerkship’ phase, but lacked a formal program in the clerkship years [34]. This may be also reflected in our study where we found students in early clinical years giving a lower rating to our program effectiveness (Table 1). It is important that schools provide a longitudinal professionalism curriculum throughout the entire MD Program supported by positive role models and school administrative activities. Students want to be inspired by guest lecturers and to celebrate exemplary professionalism by creating awards for both students and faculty. They also expect to have professionalism lapses by students, residents and faculty managed appropriately. Our students identified several improvements to strengthen professionalism programs, including guest lecturers, awards to staff, and medical program professionalism web pages (Table 4).

Based on this extensive review and students’ perspectives, we have developed an ‘Action Plan on Professionalism” which is in process of being implemented at our school (Table 5). Our program is now being modified and expanded to include more explicit teaching on professionalism in the preclerkship phase, and plans for a full four-year program accompanied by trained faculty are well underway. A Reaffirmation Ceremony with presentations from residents and faculty and a reading of an oath is held at the start of clinical clerkship, to remind the students of the attributes and responsibilities as they enter the clinical phase of their learning. The orientation week now also includes interprofessional sessions, that allow early exposure to the field of collaborative practice.

**Table 5:** Action plan on professionalism: strategies for developing student professional identity

1	A “White Coat ceremony” early in the program, with reading of a declaration or professional code.
2	Formal introduction to the attributes of professionalism.
3	Case based sessions led by trained faculty.
4	Interprofessional interaction activities.
5	A Reaffirmation Ceremony prior to entry into clerkship.
6	Development and adoption of innovative methods for feedback and development of professional identity such as Multi-source feedback and a portfolio.
7	Faculty development focused on teaching, modeling and evaluating professionalism.
8	Professionalism Awards for faculty members and medical students.
9	A clear process of dealing with professionalism concerns (for students, residents and faculty), including clear consequences and remediation plans.
10	A conscious effort to address the learning environment/the hidden curriculum by all those involved in formation of the student identity.

Recent work on reflection includes studies that show that student narratives can influence professional development, as debriefing allows individuals to explain, analyze and synthesize information and emotional states, and to improve performance in similar situations in the future [35]. Student narratives can also help understand the informal and hidden curriculum, and assist students in developing their professional identity [36].

An electronic portfolio on our core competencies program has been implemented over the four years, to facilitate student reflection on all the CanMEDS competencies, including professionalism [37]. This is supported by coaches, many of whom are primary care physicians, and who participate in an ongoing Faculty Development program. This has become a great venue for our students to debrief, in a supportive and safe environment, on difficult experiences in clerkship, some of which relate to lapses of professionalism. In their work Hilton and Slotnik [38] use the Greek term “phronesis”, practical wisdom that is developed by experience and reflection on the experience. It is with practice and time that students develop their professional aptitude along with skills and knowledge, a period the authors call proto-professionalism. The portfolio program can foster this process.

The limitations of this study include the fact that the survey involved only one school and may not be generalizable. We did however have information from other sources such as the Canadian Graduation Questionnaire indicating that many issues regarding professionalism are similar across the country [19]. The response rate to our survey was lower in the clerkship years, with 14% in year 3 and 34% in year 4, as many were immersed in local and remote clinical placements. Unfortunately despite frequent email reminders, and the online design, the University of Ottawa clerkship students are often out of province or the country, thus less “captive” and less available to contact. This is a loss of potentially valuable information from students who could have helped to inform on many areas, particularly regarding the learning environment in clerkship. The survey was conducted in 2006 and since then many of the schools have implemented new initiatives for the professionalism curriculum, so it is possible that some of the challenges exposed by this study have been addressed.

## Conclusions

Future studies will need to determine the outcomes of our newly implemented program, ideally evaluations that include focus groups and in-depth interviews with key stakeholders. This will include the formal curriculum, the administration response to lapses, and the learning environment. Change is required to address the needs of our learners and our society. The University of Ottawa model may be of value to other schools or institutions involved in teaching medical students. Medical students are vulnerable in their path to attaining all the attributes required of a professional. In this journey they are often exposed to and socialized to medical milieus that increasingly are very dispersed with the increase in distributed and integrated medical curricula, which may include community and rural placements. It is vital that all those who serve as role models are aware of the fundamental principles and challenges in attaining and maintaining excellence in professionalism competency. As the teachers and role models within our self-regulated profession it is our duty to take ownership of the task of developing the professionalism of our students, before someone else does [20].

### Key points

1. Professionalism must be central to the MD Program curriculum and be integral to the evaluation of learners and teachers.

2. Role modeling of professionalism in all settings (including hospitals, clinics and other clinical milieus) is identified by students as the key component of a positive learning environment.

3. Faculty Development for all teachers in health care must focus on activities dedicated to excellence in role modeling of professionalism attributes (including empathy, respect, continuous self-improvement and altruism).

## Competing interests

The authors declare that they have no competing interests.

## Authors’ contributions

All the authors have made significant contributions to the article. Anna Byszewski and Walter Hendelman led the conception and design of the study, acquisition of the data and data analysis, and drafted and revised the article. Caroline McGuinty was involved in the data interpretation, literature review and drafting of the article. Genevieve Moineau assisted with survey design, data interpretation and revisions of the article. All authors gave final approval of the version to be published.

## Pre-publication history

The pre-publication history for this paper can be accessed here:

http://www.biomedcentral.com/1472-6920/12/115/prepub
